# Microfluidic Based Fabrication and Characterization of Highly Porous Polymeric Microspheres

**DOI:** 10.3390/polym11030419

**Published:** 2019-03-05

**Authors:** Benzion Amoyav, Ofra Benny

**Affiliations:** The Institute for Drug Research, The School of Pharmacy, Faculty of Medicine, Campus Ein Kerem, The Hebrew University of Jerusalem, Jerusalem 9112192, Israel; amoyav@gmail.com

**Keywords:** porous microspheres, porosity, PLGA, PLA, tissue engineering, microfluidics, focused-flow

## Abstract

Polymeric porous particles are currently used for various applications in biotechnology, tissue engineering and pharmaceutical science, e.g., floating drug delivery systems and inhaled formulations. Particle shape and size depend on variable parameters; among them, polymer type and concentration, stirring speed, pH and type of solvent. In this study, porous poly(lactic-*co*-glycolic) acid (PLGA) and poly(d,l-lactide) (PLA) microspheres (MPs), with varying sizes and morphologies, were synthesized and optimized using both batch formulation and a flow-focusing microfluidic device. A well-established method of preparation utilizing solvent evaporation and the double emulsion technique was performed. Similar to other batch encapsulation methods, this technique is time and reagent consuming and consists of several steps. Hence, although porous structures provide tremendous opportunity in the design of new applications for tissue engineering and as improved controlled-release carriers, the synthesis of these particles with predefined properties remains challenging. We demonstrated the fabrication of porous MPs using a simple microfluidic device, compared to batch synthesis fabrication; and the effect of solvent, polymer concentration and type, post-hydrolysis treatment, on porosity degree. Moreover, a kinetic release study of fluorescent molecule was conducted for non-porous in comparison to porous particles. An overview of future prospects and the potential of these porous beads in this scientific area are discussed.

## 1. Introduction

Biodegradable polymeric porous microspheres (MPs) are characterized by unique morphologies that have shown a wide range of biomedical applications in tissue regenerative medicine, as cell culture scaffolds and as drug-controlled release carriers [1]. Porous MPs are featured with large surface area and low density, and this confers a particular profile of drug absorption and release kinetics. Controlling these parameters makes these particles effective candidates for drug delivery and for use in various biomedical applications [2]. Regarding drug carriers, the delivery of controlled release active pharmaceutical ingredients such as proteins, inhaled steroids, antibiotics and anti-cancer drugs is critical for treating many diseases; this is due to several advantages in the formulation of controlled delivery and improved targeting [3,4]. For tissue engineering and regenerative medicine, porous material provides a three-dimensional (3D) cell-attached scaffold that mimics physiological conditions of the natural extracellular matrix to support cell differentiation and proliferation [5,6,7]. Poly(lactic-*co*-glycolic) acid (PLGA), poly(d,l-lactide) (PLA) and polycaprolactone (PCL) polymers have well-established safety profiles and serve as biomaterials for many FDA-approved products. Their excellent biocompatibility and biodegradability profiles and good mechanical properties make them attractive materials for drug delivery and implants [8,9,10]. These features are controlled by various parameters, such as molar mass and copolymer chain ratio, and hence, render them commonly used and ideal materials for various biomedical and pharmaceutical applications [11]. Yet, the fabrication of monodisperse polymeric drug particles is a central challenge in the development of new and advanced drug delivery systems. In contrast to traditional batch encapsulation techniques, the rising technology of microfluidics enables controlling fluid flow on a microscopic scale, and the production of monodisperse micro to nanoparticles [12,13]. Downscaling processes with a microfluidic device offers many advantages including low cost, fabrication of uniform droplets, high throughput and reproducibility [14,15]. Among the diverse usages, one application of microfluidic devices is the synthesis of emulsions for biotechnological industries, medicine and even cosmetics [16,17]. In our previous work, we demonstrated the production of micro- and nano-particles using varying conditions in a single simple chip design [18].

To date, several techniques have been demonstrated for fabricating porous MPs. These include conventional methods (e.g., seeded polymerization, emulsion evaporation) and new methods such as microchannel emulsification. For example, biodegradable polymeric MPs were used as injectable scaffolds. However, in this method, the average MP diameter was quite dispersed, with limited control over porosity [19]. Making porous PLGA beads was also possible using a glass capillary device based on water-in-oil (W/O) emulsion of homogenized gelatin as a porogen [20]. However, in addition to the low porosity properties of this method, the fabrication time was dramatically prolonged, and the product was exposed to heat in order to remove the gelatin remnants. Microfluidic based methods were shown to provide better control over the microenvironment of 3D scaffolds of calcium alginate, for studying the response of cells to various conditions in their environment [21]. Accordingly, biodegradable porous structures are expected to be optimal microcarriers for 3D cell cultures, tissue engineering and drug delivery. Furthermore, the integration of microfluidic technology with polymeric scaffolds and 3D cell cultures seems to offer a promising platform for in vivo-like organ and tissue engineering. However, most of these production protocols are based on conventional, emulsion-based batch methods. Here, we aimed to display the integration of a microfluidic platform in this field and to provide an optimal procedure for porous MP particles, both in a batch method as water-in-oil-in-water (W/O/W) emulsion, and with a microfluidic flow-focused chip. Polymeric porous MPs were fabricated using varying types of organic solvents, polymer types and concentrations. Moreover, the post-hydrolysis treatment by sodium hydroxide solution was implemented in addition to changes in fabrication methods. Finally, a kinetic release study of 6-coumarin, comparing porous and non-porous MPs, was carried out to evaluate the influence of porosity on drug-like molecule release. 

## 2. Materials and Methods

### 2.1. Materials

Poly(lactic-*co*-glycolic) acid 50:50 (PLGA molecular weight (*M*_w_) 40,000–75,000, Sigma-Aldrich, Steinheim, Germany), PLGA 75:25 (76,000–115,000 *M*_w_, Sigma-Aldrich), poly(d,l-lactic acid) (PLA *M*_w_ 75,000–120,000, Sigma-Aldrich), dichloromethane (DCM, Bio-Lab, Jerusalem, Israel), ethyl acetate (EA, Bio-Lab), chloroform (CF, Bio-Lab), polyvinyl alcohol (PVA *M*_w_ ~67,000, Sigma-Aldrich), double distilled water (DDW), polycaprolactone (PCL, average *M*_n_ ~14,000 and 80,000, Sigma-Aldrich), ammonium bicarbonate (ABC, NH_4_HCO_3_, Sigma-Aldrich), sodium hydroxide (NaOH, Sigma-Aldrich), 6-coumarin (Sigma-Aldrich).

### 2.2. Microfluidic System and Chip

**Figure 1:**
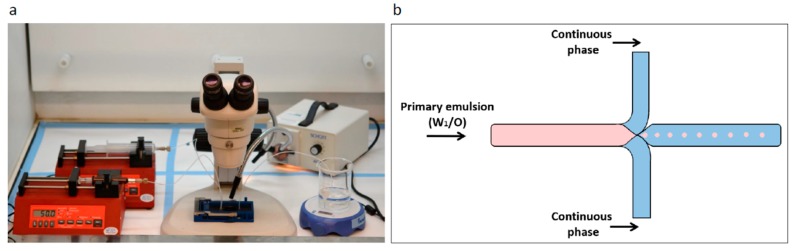
Microfluidics system for preparation of porous microspheres (MPs) with varying properties. (**a**) Laboratory microfluidic flow system set up. Two syringe pumps precisely control the fluid volume and flow rate injected through the chip. Droplets are collected in the outlet into a stirred glass cup. (**b**) Schematic illustration of an enlarged junction in the focused-flow chip design used for MP synthesis. The flow through the orifice enables a controlled droplet break-up, which is required for yielding monodisperse MPs.

The microfluidic system used was from Micronit Microtechnologies (Enschede, Netherland). The chip is made of durable borosilicate glass and the fluidic slide is made of polypropylene, its dimensions are 45 mm × 15 mm; channel width and depth are 100 and 20 µm, respectively. Experiments were performed using the flow-focused design (Figure 1).

### 2.3. Preparation of Water-in-Oil Emulsion

**Figure 2:**
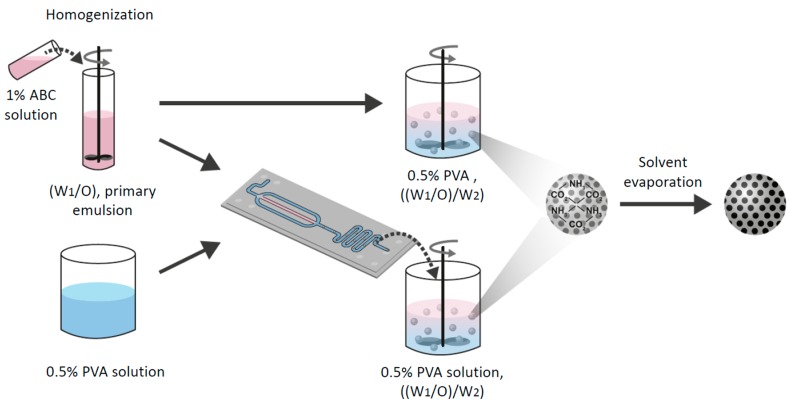
A schematic illustration of the preparation method of porous MPs using either “batch” or the microfluidic technique. Ammonium bicarbonate (ABC) 1% solution with polymer solution was homogenized to form a primary emulsion (W_1_/O). Then, the emulsion was introduced to either a vessel of 0.5% (*w*/*v*) polyvinyl alcohol (PVA) solution or into a microfluidic droplet generation chip. Finally, the secondary double emulsion ((W_1_/O)/W_2_) was stirred with an overhead propeller to ensure complete evaporation that forms porous solid MPs.

Porous MPs were prepared by the double emulsion method or via a microfluidic flow-focused chip design. Briefly, a given amount of polymer was dissolved into a non-polar solvent (e.g., DCM, CF) or a polar solvent (e.g., EA). Two ml of 1% *w*/*v* ABC aqueous solution were added to the polymer solution. This mixture was homogenized with MICCRA homogenizer disperser D-9 (Heitersheim, Germany) at 11,000 rpm for 3 min to form the primary emulsion (W_1_/O). Then, the primary emulsion was introduced to either a vessel of 0.5% (*w*/*v*) PVA solution or a microfluidic droplet generation chip (Figure 2).

#### 2.3.1. Porous Microspheres using Microfluidics

W_1_/O emulsion was formed as detailed above and the primary (W_1_/O) emulsion was gently perfused into the microfluidic flow-focused chip using a glass syringe. The flow-focused chip design consisted of a cross junction, where the primary emulsion (W_1_/O) entered through a central channel and was squeezed at the orifice by a continuous aqueous phase of 0.5% (*w*/*v*) PVA solution to form a controlled droplet break-up of the secondary emulsion ((W_1_/O)/ W_2_). The double emulsion was stirred with an overhead propeller at 600 rpm for 4 h to ensure complete evaporation of the organic solvent. The MPs were washed with DDW and centrifuged at 3000 rpm for 2 min to eliminate adsorbed PVA. Subsequently, the washed MPs were immersed in an aqueous NaOH (0.2 M) solution and washed thoroughly three times with DDW to remove any NaOH residues. Finally, to prepare solidified particles, the solution of washed particles was frozen overnight in −80 °C and lyophilized (Freezone 6 plus, Labconco, Kansas city, MO, USA) to produce a dry powder of particles that was stored at −20 °C.

#### 2.3.2. Porous Microparticle Preparation using the Batch Method

The primary emulsion (W_1_/O) was instantly poured to 250 mL of 0.5% (*w*/*v*) aqueous PVA solution with an overhead propeller, stirring at 600 rpm for 4 h to allow evaporation of the solvent from the secondary emulsion ((W_1_/O)/ W_2_) to form hardened MPs. The steps previously described to produce the final MPs were followed.

### 2.4. Particle Characterization-Electron Microscopy

The morphology of MPs was characterized and imaged using a scanning electron microscope (SEM). For SEM (FEI Quanta 200 microscope) analysis, a small amount of the samples was spread on a conductive adhesive carbon tape attached to a SEM grid and a thin film of Pd/Au coating sputtered onto the sample (SC7620 Spatter coater, Laughton, East Sussex, UK). The mean diameter of particles was calculated based on the measurements of 100 randomly chosen particles using ImageJ program (Rasband, W.S., ImageJ, U.S. National Institutes of Health, Bethesda, MD, USA, https://imagej.nih.gov/ij/, 1997–2018).

### 2.5. Confocal Microscopy Imaging

A Nikon A1R confocal light microscope (NY, USA) was employed to assess the core−shell structures of the porous MPs. To optically visualize the porous structure, a green dye (6-coumarin) was dissolved with the polymer primary solution to obtain fluorescent MPs. Volume of 300 µL of hardened particles were placed on a glass slide. The 6-coumarin dye was excited at 488 nm.

### 2.6. Porosity

The degree of porosity of the MPs was calculated based on the weight ratio of porous MPs to non-porous MPs characterized with same average diameter. The MPs filled to top in a 0.2 mL minitube and tapped 100 times before their weight measured by analytical grade scale. To achieve statistically significance, this calculation carried out with 10 repetitions in each tested group.

### 2.7. In Vitro Release Kinetics Study

The release kinetics profiles were determined for 6-coumarin that were loaded in porous MPs fabricated with the microfluidic technique. For comparison, non-porous 6-coumarin loaded MPs of similar mean size were also prepared using microfluidic technique. 30 mg of MPs with a mean size of 100 μm were inserted into a dialysis membrane bag (*M*_w_ cutoff = 25 kDa, Spectra/Por Biotech Regenerated Cellulose, VWR) against 50 mL PBS 0.1% tween 80 solution (pH = 7.4) in a release glass bottle with mild magnetic stirring of 100 rpm, at T = 37 °C. At predetermined time points, samples of 200 μL were collected from the external solution and immediately measured at Ex/Em 480/530 wavelength using a plate reader (Synergy HT Multi-Mode Microplate Reader, Bio Tek, Winooski, VT, USA). Immediately after measuring, the 200 µL were returned to the bottle. The percentage of release was calculated by normalizing the obtained data at each time point with the cumulative total amount. The release tests were conducted in triplicate.

### 2.8. Statistics

The experiments were performed with n = 3–4. All data measurements were represented as means ± standard deviations (SDs). To identify statistically significant differences between groups, student’s t-test was used. One-way ANOVA followed by Tukey’s test for post-test comparisons was used when more than two groups were compared. Probability values of p < 0.05 and p < 0.01 were considered significant. 

## 3. Results and Discussion

### 3.1. The Effect of Polymer Concentration

**Figure 3:**
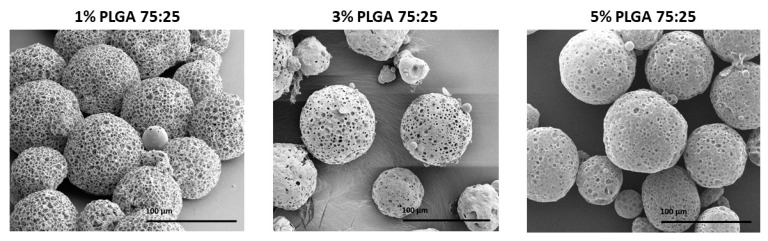
Scanning electron microscope (SEM) images of poly(lactic-*co*-glycolic) acid (PLGA) porous microsphere particles fabricated by batch synthesis with increasing PLGA 75:25 concentrations (% *w*/*v*), 1%, 3% and 5%. The scale bars are 100 μm.

Viscosity of the organic phase was shown to have a great effect on the final porous morphology and stability [22]. To determine the effect of polymer viscosity on the characteristics of MPs, a series of polymer concentrations in DCM solutions was tested. The concentration of PLGA in the organic phase demonstrated a substantial effect on the final morphology. As shown in Figure 3 and Table 1, increasing polymer concentration from 1% to 3% and 5%, reduced the porosity degree of the particles from 83% to 71% and 64%, respectively, without conferring a statistically significant difference in the mean diameter of the MPs (88 ± 2 μm).

The experiment with 1% (*w*/*v*) PLGA 75:25 yielded MPs characterized with the highest porosity degree and were found to be statistically significant compared to the other two formulations. The increased porosity can be explained by the fact that a higher polymer concentration yielded a viscous polymer solution [23]. This provided more massive polymer-polymer interactions and a strong interfacial tension solution, and hence, created a denser porous network. In addition, increased viscosity may reduce the ability of the aqueous porogen gas bubbles to penetrate through the primary emulsion microdroplets to the membrane of the surface shell. Thus, a lower polymer concentration solution could result in the formation of a more porous and visibly interconnected scaffold of the MPs [24].

### 3.2. The Effect of Polymer Type and Molecular Weight

**Figure 4:**
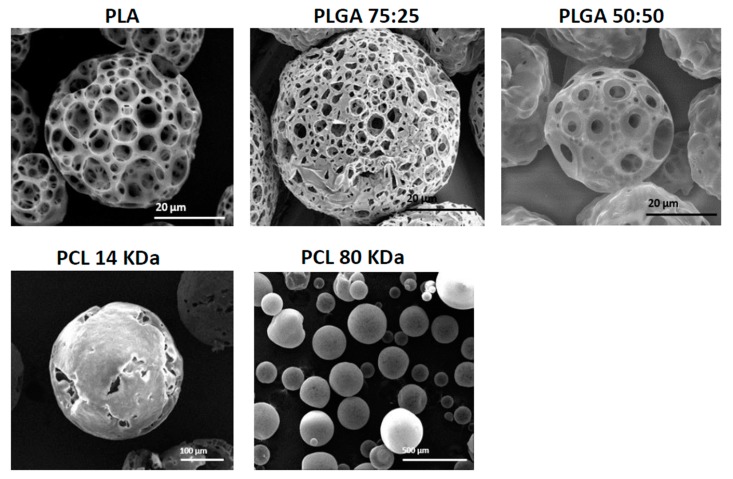
SEM images showing the morphology of porous and non-porous microspheres obtained with different polymers and molecular weights using batch synthesis. PLA, PLGA 75:25, PLGA 50:50, PCL 14 kDa and PCL 80 kDa.

For compatibility with human functions, porous MPs should be fabricated from non-toxic, biocompatible and biodegradable polymers. In this study, we compared three polymers that are currently used in FDA approved biomedical products—PLA, PLGA (50:50 and 75:25) and PCL (14 and 80 kDa). In these experiments, all MPs were fabricated under the same conditions, using the batch technique with 2% (*w*/*v*) polymer dissolved in DCM solution. Figure 4 shows the results of fabricated MPs with the above-mentioned polymers. When PLGA 75:25, 50:50 and PLA polymers were used, porous MPs with mean diameters of 83 ± 1, 34 ± 1 and 62 ± 2 μm, respectively, were obtained. PCL 1 kDa led to the formation of hollow spheres with cracked slits on their shell layer, with mean size of 310 ± 3 μm. On the other hand, the formulation with PCL 80 kDa produced sphere particles with mean size of 107 ± 3 μm. In general, during MP formation, a phase inversion process yields a microporous structure in the core and at the surface layer. From both PLGA and PLA polymers, porous MPs were obtained; however, they differed in their surface pore size. The pore size in PLA particles was 13 ± 6μm and in PLGA, 5 ± 2 μm. This difference may be attributed to differences in *M*_w_, and to their hydrophobicity and hydrophilicity, respectively. *M*_w_ and the degree of crystallinity are major factors that determine such properties of polymers as mechanical strength, viscosity and solubility [25]. PLGA is characterized by reduced crystallinity compared to PGA, when co-polymerized with PLA, and thus, PLGA increases the rate of polymer chain hydration [8,11]. This may lead to an enlarged ABC micro-bubble content in each droplet, which produces carbon dioxide (CO_2_) and ammonia (NH_3_) gas bubbles, and subsequently leads to the formation of a more porous network shell. Moreover, as polymer glycolic acid content increased, the surface pore size decreased. This may be attributed to polymer hydrophilicity [7], which results in relatively low solubility with the DCM phase, and hence, small pore size. PLA, which is more hydrophobic than PLGA polymers, may absorb less water and consequently form larger pores in its surface shell net. Interestingly, PCL in our experimental conditions did not produce a porous sphere. This result could be attributed to the high hydrophobicity of PCL, which results in a lower concentration of ABC microdroplets that can be homogenized in the primary emulsion (W_1_/O) droplets. Hence, a non-porous particle structure is obtained, due to the relative instability of the W_1_/O emulsion.

### 3.3. The Effect of Solvent Type

**Figure 5:**
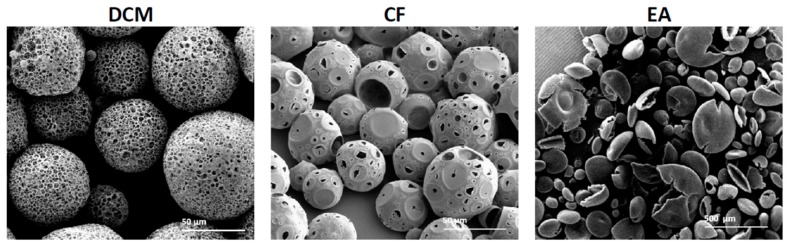
SEM images of PLGA (75:25) particles obtained by batch synthesis show the influence of organic solvent on microsphere formation and porosity with three solvents: dichloromethane (DCM), chloroform (CF) and ethyl acetate (EA).

Polymeric MP morphology is affected by the type of organic solvent [26]. We compared the fabrication of porous spheres using three solvents: two non-polar solvents, DCM and CF, and one polar solvent, EA. The particles with the best homogenous porosity characteristics were prepared with DCM and CF as can be seen in Figure 5 and Table 2.

When EA was used as a solvent with the primary emulsion, non-porous empty core spheres were obtained, which were characterized by flat erythrocyte-like structures. DCM and CF led to the fabrication of MPs with spherical particles and porous shells. We attributed these morphological changes to the polarity and miscibility of the solvents in the aqueous phase, and hence the evaporation rate [27]. To incorporate pores into an MP shell, the porogen aqueous solution should be immiscible with the dissolved polymer solution. The relatively high solubility of EA in water compared to non-polar solvents results in a faster removal rate compared to the non-polar solvents. This difference may lead to a high osmotic variation between the microdroplets and the outer aqueous phase, which then causes coalescence of inner aqueous micro-droplets to form an erythrocyte-like morphology [28]. The non-polar solvents are characterized by lower solubility with the aqueous inner phase, which results in a slow and gradual removal process. Subsequently, the inner aqueous droplets of the primary emulsion can develop into a porous structure, which ultimately forms porous MPs. Although porous spheres were obtained with non-polar solvents, differences existed between them. Particles fabricated with DCM exhibited a denser porosity structure with lower porosity degree compared to particles fabricated with CF (78% and 93%, respectively). Moreover, a larger average diameter obtained with DCM compared to CF particles (150 and 40 μm, respectively). These differences may be attributed to the solubility of the non-polar solvents in the aqueous phase. The lower solubility of CF than DCM in water [27,29] may lead to a relatively fast diffusion process and to the formation of MPs that are characterized by a small diameter and large pores with overall higher porosity degree (Figure 5).

### 3.4. The Effect of the Synthesis Method

**Figure 6:**
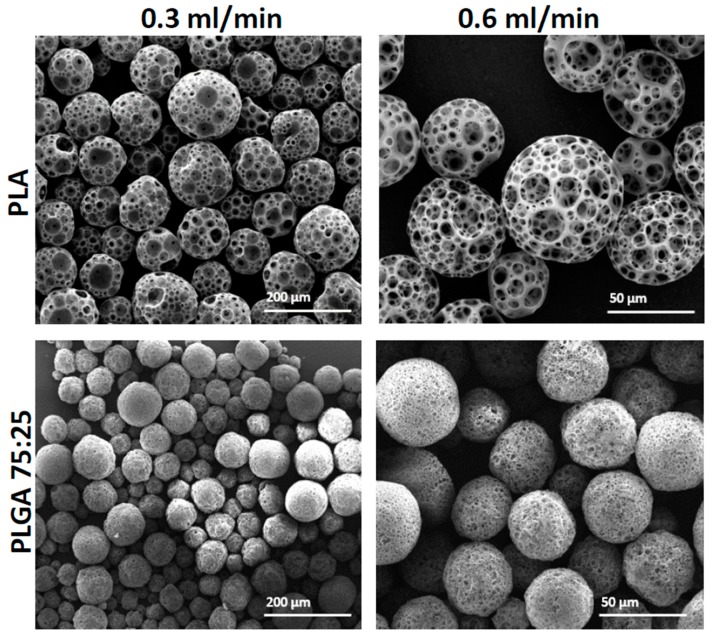
SEM images of porous polymeric MPs fabricated with increased continuous phase flow by a microfluidic droplet generation device. PLA and PLGA 75:25 MPs were fabricated with either 0.3 mL/min or 0.6 mL/min 0.5% PVA continuous phase. The primary emulsion (W_1_/O) flow rate was kept constant at 0.05 mL/min.

By using a microfluidic platform, we can gently control the flow rate of the two joined phases: the primary emulsion (W_1_/O) and the continuous (W_2_) 0.5% (*w*/*v*) PVA phase. In addition to their effects on flow rate and particle size, channel geometry and phase viscosities can influence the fabricated droplet size [12]. Compared to the conventional “batch” synthesis, the ability of microfluidic devices to manipulate micro and nano-liter volumes of liquid and to control the mixing process [30] serves as an excellent tool for generating reproducible droplets and particles characterized with a narrow size distribution. During the fabrication process, the rate of the dispersed primary emulsion (W_1_/O) phase remained constant at 0.05 mL/min, while the continuous PVA 0.5% *w*/*v* flow rate was set to either 0.3 or 0.6 mL/min. As shown in Figure 6, in these flow rate conditions, by using PLA and PLGA 75:25 polymers, the mean MP diameters obtained were 112 ± 4 μm and 53 ± 7 μm for PLA, with flow rate 0.3 and 0.6 mL/min, respectively; and 77 ± 4 μm and 43 ± 5 μm for PLGA, with flow rate 0.3 and 0.6 mL/min, respectively. The increased continuous flow rate, relative to the primary emulsion (W_1_/O) flow rate, resulted in decreased droplet size and consequently smaller MPs. This is due to a high rate of droplet cutting by a pincer movement at the orifice region (Figure 1b). Accordingly, a decrease in flow rate would lead to an increase in droplet size.

As continuous phase flow rate increased, the degree of porosity of the fabricated MPs increased as well. This may be due to the increased shear stress applied on each droplet, which correspond with the increased flow rate [31,32]. The upshot could be increased evaporation of gas bubbles, and the formation of a more porous structure. On the other hand, when we used the microfluidic platform, we obtained MPs that were characterized by a narrow size distribution. This demonstrates one of the main advantages of using the microfluidic platform for porous bead generation. Nonetheless, this process has its natural limit. When the dispersive phase flow rate is too slow relative to the continuous phase, droplets are not produced due to back-flow pressure of the aqueous phase through the orifice. In addition to the effect of size, another difference that could be discerned from batch synthesis was the effect on porosity level (Table 3).

MPs fabricated with PLGA polymer exhibited lower average porosity degree compared to MPs fabricated with PLA (74% and 83%, respectively). This result found to be statistically significant and could be referred to level of hydrophobicity. PLA polymer characterized with a higher hydrophobicity compared with the glycolic acid containing PLGA polymer. This may lead to a decrease in the number of water droplets dispersed in the oily phase and possibly the formation of less dense porous net, and hence a larger pore formation.

### 3.5. The Effect of NaOH Hydrolysis of the Microspheres

**Figure 7:**
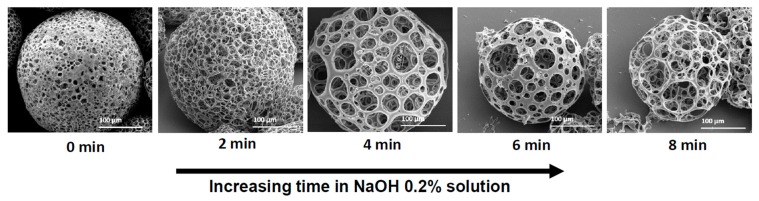
SEM images of 3% (*w*/*v*) PLGA 75:25 porous microspheres obtained by the microfluidic technique at increasing soaking time in NaOH 0.2% solution. As immersion time increased, a more porous structure was obtained.

During MP preparation, either by the batch or the microfluidic method, we found that the porosity level of the fabricated particles was not homogeneous and was hard to control. We assume this was due to the small micro gas-bubbles struggling to penetrate from the core along the entire distance to the MP surface layer. Therefore, to modify a desired porous morphology, we immersed the MPs in 0.2% NaOH solution for specific time periods. Figure 7 shows the morphologies of MPs obtained with increased exposure time to NaOH solution. As we increased the hydrolysis time, larger pores were obtained. Furthermore, it can be seen in Table 4 as the immersing time increased from 0 to 8 min, the porosity degree of the MPs increased as well from 71% to 98%, respectively.

This alkaline solution facilitates hydrolysis, and hence, causes gradual exposure of the porous scaffold [33,34,35]. After this controlled hydrolytic process, the porous morphology of the MPs regarding their scaffolds was more homogeneous in both the interior and surface layers. Moreover, prolonging the hydrolysis from 2 to 4 min increased the porosity without modifying the MP diameter. When we increased the time to 6 and 8 min, in addition to increased porosity degree (91% and 98%, respectively), the diameter of the MPs decreased by ~80 μm, from 306 ± 1 μm to 223 ± 7 μm on average. This indicates that exposure time to basic conditions may have an optimum level for both porosity and diameter. Time-controlled hydrolysis was found to be a simple method to control porosity features of the fabricated MPs compared to the modified polymer concentration. Moreover, this hydrolysis process could serve as a good method for increasing MP hydrophilicity by exposing carboxylic groups on the interior and the surface layer, and hence increasing the likelihood of adsorbing hydrophilic molecules. However, the delicate balance required in this hydrolysis treatment should be emphasized. Accordingly, as we increased the exposure time in the NaOH solution, we increased the porosity, and this could lead to a substantial decrease in mechanical particle strength.

### 3.6. The Effect of Porosity on the Kinetics Release Profile

Drug release of polymeric carriers depends on varying factors; among them, polymer type and composite, polydispersity index, drug solubility and other formulation additive properties [11,36].

**Figure 8:**
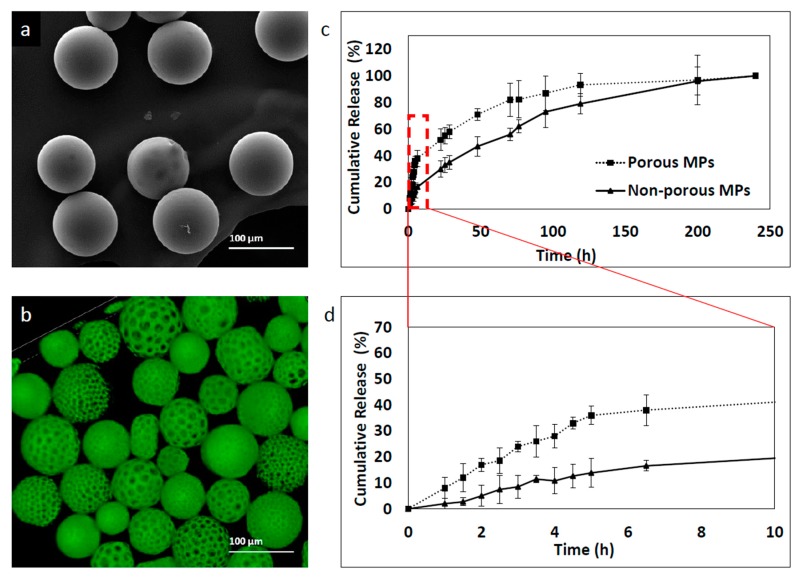
6-coumarin release kinetic profile of MPs fabricated with the microfluidic approach. Samples were collected at a number of points of time and fluorescence was measured by a plate reader. Empty particles were used as control references. (**a**) Non-porous MPs, (**b**) porous MPs, (**c**) cumulative release profile of 250 h and (**d**) cumulative release profile of the initial 10 h.

To determine the effect of porosity on MP release kinetics, two formulations of porous and non-porous MPs were prepared using the microfluidic technique with 0.8 and 0.05 mL/min of continuous and dispersed phase flow rates, respectively. Six-coumarin was used as a model hydrophobic drug. Labeled MPs were prepared at 30 mg/mL concentration of PLGA 75:25 and a mean size of 93 ± 11 μm was obtained. Although the two formulations were characterized by similar mean size, they exhibited significantly different release profiles. First, the non-porous particles showed a more controlled release profile and second, they showed a smaller “burst effect”-like profile compared to the porous micro MP group, as shown in Figure 8. The porous MPs demonstrated faster release, with a “burst effect” within the first 5 h. In contrast, non-porous MPs showed a slower release rate within the first 5 h, with almost 40% less 6-coumarin release. This phenomenon might be explained by differences in surface topography. The higher surface area of the porous compared to the non-porous MPs may lead to a faster release rate since porosity and roughness enhance the diffusion of solvents into the polymer matrix [37,38]. After ~150 h, both formulations reached a plateau zone, which continued until the end of the measurements, at 245 h.

## 4. Conclusions

In this study, polymeric porous MPs were fabricated by regulating and varying a number of key parameters by batch or by a flow-focusing microfluidic device. The MPs were fabricated with three solvents: DCM, CF and EA. The non-polar, poor water-soluble solvents (DCM, CF) successfully formed porous particles with varied porosity degree; in contrast, EA, a polar water-soluble solvent failed to produce any such particles. This difference was attributed to differences in miscibility and viscosities in the aqueous solutions and hence in evaporation rate. Post-hydrolysis treatment with 0.2% NaOH solution was found to be a compatible method to control the degree of particle porosity. The degree of porosity of MPs was quite broad, ranging from 68% to 98%, and thus provide an additional degree of freedom for rational design of porous structure. A treatment of 2 to 4 min was identified as optimal for increasing porosity level by 25% without changing the MPs initial diameter. This enabled optimization of the MP properties compared to adjustments in polymer concentration. To determine the effect of polymer type, three biocompatible polymers (PLA, PLGA and PCL) were tested under the same fabrication conditions. Viscosity, hydrophobicity, hydrophilicity and M.W. are main characteristics of the polymer that govern porosity level and the size of particle formation. With PLGA and PLA, the morphology and porosity properties of the particles obtained were optimal. In contrast, when PCL was used, hollow particles were obtained, characterized by cracked slits on their shell layer. We attributed this to the high hydrophobicity and low solubility of aqueous ABC in the primary droplet emulsion. Microfluidic based fabrication was found to be a convenient way to control particle size and porosity degree via gradually variation of flow rates. Moreover, the fabricated MPs exhibited a higher precision, with a narrow size distribution compared to that obtained by the batch traditional method. An in vitro kinetic release study revealed that the porous structure accelerates the degradation of the MPs and the release of 6-coumarin from its matrix, compared to a non-porous particle. This was attributed to high surface area of the porous matrix, which enhances the diffusion of the molecule to the inspected medium. The potential of this highly versatile method for porous polymeric MPs in the use of drug delivery or tissue engineering is tremendous. Overall, these studies demonstrated that highly porous polymer MPs can be successfully achieved by DCM or CF, with or without post-hydrolysis treatment, and that further fine-adjustments are feasible using a microfluidic platform. Future studies should consider and optimize additional parameters; in addition, mechanical strength with respect to morphology and porosity should be determined. Another aspect that should be explored in future studies is the effect of MPs characterized with different diameters and porosity degrees on release kinetics profile. Furthermore, additional microfluidic based protocols for advanced polymeric carrier synthesis should be developed to increase the range of formulations obtained. Finally, we believe that the ability to fine tune porosity while achieving ideal and predetermined properties will pave the way to novel applications in various biomedical and engineering fields.

## Figures and Tables

**Table 1:** Structure characterization of PLGA 75:25 microspheres obtained with increased polymer concentration, *p* < 0.05. The average diameters and porosity (%) were calculated based on SEM images with image analysis software ImageJ and weight ratios as depicted in Section 2.6.

Factor	PLGA 75.25 Concentration (% *w*/*v*)		
	1%	3%	5%
Average diameter (μm)	91 ± 1	86 ± 2	87 ± 3
Average surface pore diameter (μm)	11 ± 3	10 ± 4	8 ± 3
Porosity (%)	83.7	71.3	68.4

**Table 2:** Structure characterization of PLGA 75:25 microspheres obtained with three different solvent types, DCM, CF and EA. *p* < 0.05. The average diameters and porosity (%) were calculated based on SEM images with image analysis software ImageJ and weight ratios as depicted in Section 2.6.

Factor	Type of Solvent		
	DCM	CF	EA
Average diameter (μm)	150 ± 6	41 ± 4	-
Average surface pore diameter (μm)	6 ± 2	8 ± 6	-
Porosity (%)	78.4	93.3	-

**Table 3:** Structure characterization of PLGA and PLA microspheres obtained in microfluidics method with increased flow rates regimen, *p* < 0.01. The average diameters and porosity (%) were calculated based on SEM images with image analysis software ImageJ and weight ratios as depicted in Section 2.6.

Factor	Type of Polymer (Flow Rate in mL/min)			
	PLGA 75:25 (0.3)	PLGA 75:25 (0.6)	PLA (0.3)	PLA (0.6)
Average diameter (μm)	77 ± 4	43 ± 5	112 ± 4	53 ± 7
Average surface pore diameter (μm)	4 ± 7	3 ± 8	23 ± 8	12 ± 5
Porosity (%)	71.3	78.3	81.5	86.6

**Table 4:** Structure characterization of PLGA 3% (*w*/*v*) microspheres obtained with increasing immersing time in NaOH 0.2% solution, *p* < 0.01. The average diameters and porosity (%) were calculated based on SEM images with image analysis software ImageJ and weight ratios as depicted in Section 2.6.

Factor	NaOH 0.2% Solution Immersing Time (min)				
	0	2	4	6	8
Average diameter (μm)	298 ± 11	307 ± 6	312 ± 19	238 ± 7	225 ± 14
Average surface pore diameter (μm)	8 ± 3	14 ± 5	32 ± 12	31 ± 9	53 ± 13
Porosity (%)	68.3	79.3	84.7	91.8	98.6
